# Cacao (*Theobromacacao* L.) climate zones and its associated agrobiodiversity in Arauca, Colombia

**DOI:** 10.3897/BDJ.11.e112771

**Published:** 2023-11-28

**Authors:** Carlos Gonzalez-Orozco, Mario Porcel, Sebastian Escobar, Daniel Bravo, Yeisson Gutierrez Lopez, Roxana Yockteng, Fabrice Vaillant, Margareth Santander, Sandra Llano, Karla Parra, Esperanza Briceño, Jhony Carmona, Shirley Torres, Ramiro Contreras, Andres Mendez Otero, Allende Pesca, Gustavo Araujo Carrillo

**Affiliations:** 1 Centro de Investigación La Libertad. Corporación Colombiana de Investigación Agropecuaria (AGROSAVIA), Km 17 vía Puerto López, Meta, Villavicencio, Colombia Centro de Investigación La Libertad. Corporación Colombiana de Investigación Agropecuaria (AGROSAVIA), Km 17 vía Puerto López, Meta Villavicencio Colombia; 2 Instituto de Investigación y Formación Agraria, Pesquera, Alimentaria y de la Producción Ecológica (IFAPA), Centro Málaga, Cortijo de la Cruz s/n, 29140, Churriana, Malaga, Spain Instituto de Investigación y Formación Agraria, Pesquera, Alimentaria y de la Producción Ecológica (IFAPA), Centro Málaga, Cortijo de la Cruz s/n, 29140, Churriana Malaga Spain; 3 Centro de Investigación Palmira. Corporación Colombiana de Investigación Agropecuaria (AGROSAVIA), Process & Quality Cocoa Laboratory. Valle del Cauca, Palmira, Colombia Centro de Investigación Palmira. Corporación Colombiana de Investigación Agropecuaria (AGROSAVIA), Process & Quality Cocoa Laboratory. Valle del Cauca Palmira Colombia; 4 Centro de Investigación Tibaitatá. Corporación Colombiana de Investigación Agropecuaria (AGROSAVIA), Laboratory of Soil Microbiology & Calorimetry. Km 14 vía Bogotá – Mosquera, Cundinamarca, Bogota, Colombia Centro de Investigación Tibaitatá. Corporación Colombiana de Investigación Agropecuaria (AGROSAVIA), Laboratory of Soil Microbiology & Calorimetry. Km 14 vía Bogotá – Mosquera, Cundinamarca Bogota Colombia; 5 Centro de Investigación La Selva. Corporación Colombiana de Investigación Agropecuaria (AGROSAVIA), Km 7 Via Rio Negro-Las Palmas, Sector Llanogrande, Antioquia, Medellin, Colombia Centro de Investigación La Selva. Corporación Colombiana de Investigación Agropecuaria (AGROSAVIA), Km 7 Via Rio Negro-Las Palmas, Sector Llanogrande, Antioquia Medellin Colombia; 6 Centro de Investigación Palmira. Corporación Colombiana de Investigación Agropecuaria (AGROSAVIA), Process & Quality Cocoa Laboratory. Valle, Palmira, Colombia Centro de Investigación Palmira. Corporación Colombiana de Investigación Agropecuaria (AGROSAVIA), Process & Quality Cocoa Laboratory. Valle Palmira Colombia; 7 Centro de Investigación La Libertad. Corporación Colombiana de Investigación Agropecuaria (AGROSAVIA), Km 17 vía Puerto López, Villavicencio, Meta, Villavicencio, Colombia Centro de Investigación La Libertad. Corporación Colombiana de Investigación Agropecuaria (AGROSAVIA), Km 17 vía Puerto López, Villavicencio, Meta Villavicencio Colombia

**Keywords:** agrobiodiversity, conservation of cacao, chocolate, soils, climate, Arauca, Colombia

## Abstract

**Background:**

Cacao (*Theobromacacao* L) is one of the most relevant crops in terms of economy and social rural development in Colombia. Cacao is also an important crop due to its potential to replace illicit crops and it is related to less deforestation and preserves the biodiversity. There are several cacao districts in Colombia, one of these being Arauca. The Department of Arauca is the second largest cocoa producing region in Colombia; however, it is heavily affected by armed conflict. To raise the knowledge and technology available in the region, integrating data on the occurrence of cacao farms with climatic variables becomes a powerful socioeconomic mapping tool for maintaining agrobiodiversity and food security in the region. Consequently, this type of agrodiversity data and agroclimatic approaches help to better manage agrobiodiversity, as in the cacao region of Arauca. These tools are even more relevant in biodiverse regions, such as flooded savannahs and tropical forest ecosystems, which are currently undergoing drastic changes due to agricultural expansion and climate change. One of the knowledge gaps in Colombia´s cacao regions is that there are currently no agroclimatic maps made with a social and scientific approach. This study aimed to provide a database of the spatial distribution of cacao farms in Arauca, as well as agroclimatic maps that identify and locate cacao climate regions in Arauca. We also present a presence-only matrix consisting of twenty-six tree species, or agrobiodiversity, distributed across the study region and specifically associated with the cacao forestry systems in Arauca.

**New information:**

We present the first database of both climate and agrobiodiversity data related to cacao farms in Arauca, developed with a research and socioeconomic vision that generated a novel approach for the agroclimatic zoning of cocoa in the Arauca Region and Colombia. Using 1,538 cacao farms at the regional scale, we identified two national and six regional-scale climate and soil regions. The selection at the local scale allowed us to classify 180 cacao farms comprising nine agroclimatic clusters in Arauca. We found twenty-six tree species distributed across the cacao climate zones. This dataset and its related maps also represent the agrobiodiversity of cultivated cacao locally. This is the most complete climate and agrobiodiversity dataset of cacao farms distribution in one of the top cocoa-producing regions in the country. These outputs are crucial because they constitute a baseline for developing research in the biodiversity of agroforestry systems, pests and diseases, pollutant presence, genetics, post-harvest processing and cocoa quality and safety.

## Introduction

Cacao (*Theobromacacao* L.) is a globally important staple crop. However, tropical regions where it thrives face challenges, such as a lack of steady production and increased risks due to climate change. Cacao farmers in Colombia, a country that produces fine flavour cocoa worldwide, experience these challenges daily. The Arauca Region, in northeast Colombia and part of the Orinoco River plains, has the ideal environmental and social conditions to produce premium cacao. Over the last decade, Arauca has been recognised as the second-largest cocoa-producing area in the country and has won more than one medal at the Salon du Chocolat in Paris, France, for its fine and balanced flavour profile, characterised by speciality and distinctive aromatic notes. Research in spatial analysis for mapping the agrobiodiversity of cacao and its climate zone are necessary to provide solid, science-based support to cocoa-producing communities in Arauca.

The Cacao Arauca Regalias project is an excellent example of how regional agencies can generate impact with quality research and community work. One of the project's fundamental research questions was "Where are the climate cocoa-growing regions"? These regions would be used to plan the sampling efforts and data collection of cacao varieties and diversity. Given that the territory is vast and diverse in cacao agriculture, this article presents an approach to effectively map agrobiodiversity zones and identify the areas where data collection campaigns could be carried out in Arauca.

## General description

### Purpose

Our aim is to present a spatial database of climate and agrobiodiversity cacao farms in Arauca, along with the corresponding agroclimatic zones.

### Additional information

Our selection of cacao farms, agrobiodiversity data and climate mapping approach are based on a decision-making process discussed with a multidisciplinary team of researchers with expertise in topics such as cacao production, cacao agronomy, geospatial mapping, socio-economic models, climate suitability, suitable soils and landscape, best genetic cacao varieties and productivity and post-harvest transformation. The farm-scale dataset presents a table with the diversity of tree species associated with the cacao crop in Arauca. We reported twenty-six agroforestry species that were traditionally used as canopy shade in Arauca's cacao plantations.

## Sampling methods

### Study extent

**Selecting farms and mapping climate regions**

First, we developed various datasets of cacao farms occurrences. In the sampling description methods, more details are provided about the farms' selection process. Then, the following step involved extracting the values of environmental variables that corresponded to the geographical locations of cacao farms. Next, an online statistics calculator (https://www.statskingdom.com/pca-calculator.html) was used to generate a standard Principal Component Analysis (PCA) separately for the macro and microclimate variables. The loading values of PCA Axis 1 were utilised to create a gridded map of macro- and microclimate regions using the QGIS software (QGIS 2019). These geospatial methods enabled us to identify the Arauca cacao regions through a national scale analysis.

**Results**

**National and regional scale**

**Figure 1.**
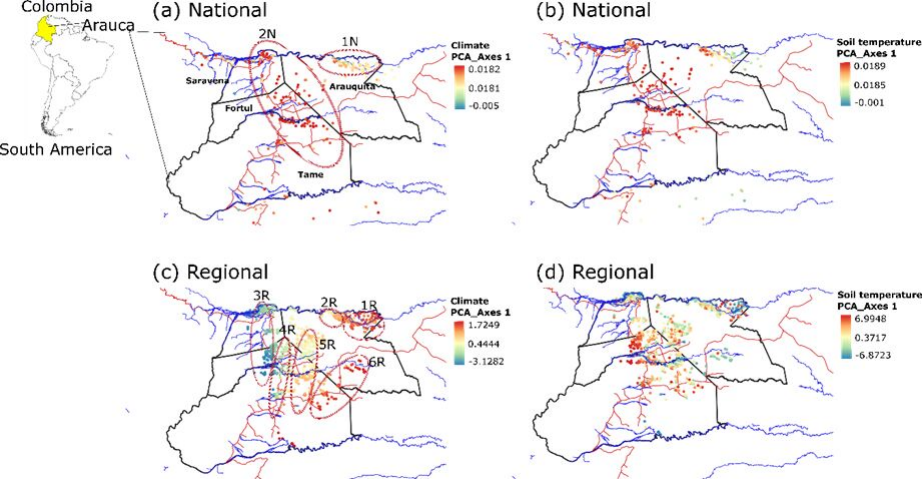
Maps of cacao climate regions in Arauca, Colombia. National (a-b) and regional (c-d) scale maps of cacao farms according to climate and soil temperature in Arauca Department, Colombia. Blue lines are rivers and red lines roads. Blue dots are indicative of negative values of the PCA, cream and red dots are indicative of positive PCA values. Cream to orange dots are indicative of mixed conditions.

We identified two national and six regional climate regions for cacao in Arauca (Fig. 1). High temperatures and low rainfall are reported on the east of the region creating a clear east to west climate pattern, as shown in Fig. 1C-D. The Arauquita Region in the northeast is hotter than the western extremes of Saravena. The foothills of Saravena, Fortul and Tame have cooler and wetter conditions. A transition zone is observed in the central region of Arauca, with mixed conditions shared by the boundaries of Saravena, Fortul and Tame. The southeast of the region, mainly covering areas of Tame, showed different conditions from the rest of the region, with a predominance of maximum temperature during the dry season and low precipitation during the wet season as key variables (Fig. 1B). A lower degree of spatial grouping was recovered, based on soil temperature regions (Fig. 1C). However, a northeast and west group of similar soil temperature conditions in Saravena and Arauquita was identified, likely influenced by the specific conditions of the Arauca riverbanks. The foothills of Fortul and Tame show similar conditions, whereas Tame seems to follow the river lines with a clear east to west gradient. Temperature seasonality shows a higher percentage of influence on the northern areas of Arauquita and Saravena, suggesting that soil temperature conditions are more variable in these areas than in the southwest foothills of Tame and Fortúl. The eastern part of Tame also seems to have a high degree of variance explained by temperature seasonality (Fig. 1D).

**Local scale**

**Figure 2.**
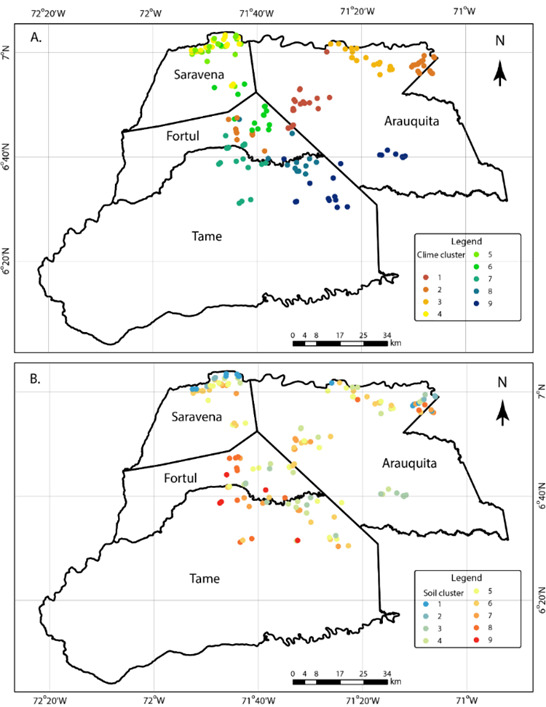
Maps of climate clusters in Arauca, Colombia. Climate (A) and soils temperature (B) clusters at local scale across the sampling area of 180 cacao farms in Arauca.

We identified nine clusters comprising 180 farms (Fig. 2). Based on the specific climate clusters (Fig. 2A), it was possible to identify the highest minimum and average temperature, wind velocity and solar radiation in clusters 2 and 3, located in the northeast of the study area. In contrast, the lowest values of these variables were identified in clusters 4, 5 and 7, which are in the northwest and southwest of the study area. Regarding rainfall, during the dry season, the lowest volumes were identified in clusters 1, 2, 3 and 9 (mainly in the west), while during the rainy season, the highest values were found in clusters 1, 6, 7, 8 and 9, in the central study area. The results of the nine soil clusters (Fig. 2B) revealed that, in the topsoil, there are clusters associated with riverbanks following floodplains (Clusters 1 to 5) and non-flooding foothills or rough plains (Clusters 6 to 9). We also considered other critical factors or cacao crop, such as the presence of cadmium in both soils and cacao beans (Bravo 2022).

### Sampling description

A national scale mapping was conducted using a previously-published occurrences dataset comprising 3,141 cacao farms in Colombia (González-Orozco and Pesca 2022). To establish the climate regions, a spatial analysis method derived from González-Orozco et al. (2014) was applied to the selected national cacao farms dataset. Seven macroclimate (Fick and Hijmans 2017) and eleven microclimate soil temperature variables at a depth of 0-5 cm were used to characterise the agrobiodiversity and the climate zones (Lembrechts 2022) of the cacao regions in Arauca. The spatial resolution of the environmental variables was 1 km^2^.

This method involved extracting the values of environmental variables that corresponded to the geographical locations of cacao farms. Next, a Principal Component Analysis (PCA) was separately conducted for the macro- and microclimate variables. The loading values of PCA Axis 1 were utilised to create a gridded map of macro- and microclimate regions using the QGIS software (QGIS 2019). These geospatial methods enabled us to identify the Arauca cacao regions through a national scale analysis.

At the regional scale, a dataset of 1,538 cacao farms in Arauca was developed (regional scale dataset). Eleven local agronomy professionals visited cacao farms in the Municipalities of Saravena, Arauquita, Tame and Fortul in Arauca. Each farm was georeferenced and uploaded to a GIS desktop. Then, the González-Orozco et al. (2014) climate mapping method was applied to the cacao farms dataset. The analysed climate variables were subdivided into temporal groups representing the dry (Nov-Feb) and wet (Mar-Oct) seasons that Arauca experiences throughout the year. The grouped macroclimate and soil temperature databases and all farms were used to map the climate and soil temperature regions using a PCA spatial mapping method (González-Orozco et al. 2014).

At the local scale, a combined effort amongst cacao farmers, agronomy field workers and a multidisciplinary team of researchers pre-selected a subset of 180 cacao farms as the best cases for future sampling efforts of cacao and other environmental variables from the 1,538 farms. The selected farms were chosen to represent good variability in climate and soil temperature conditions (Fick and Hijmans 2017, Lembrechts 2022), cadmium soil hotspots (Bravo et al. 2021), landscape types, cacao varieties and healthy cacao plantations. Other parameters, such as proximity to the road network, were also considered. Additionally, the criteria for chosing the local-scale farms included agronomic, socioeconomic, territorial and environmental traits. A second-step process was applied to classify the 180 local-scale farms selected into spatial clusters, resulting in the identification of nine clusters that were used as the sampling regions (Local-scale dataset).

A presence-only matrix of tree species (Farm-scale dataset) associated with cacao was presented as part of the agrobiodiversity assessment done across the Arauca climate zones.

## Geographic coverage

### Description

Department of Arauca, Colombia. Municipalities: Saravena, Fortul, Tame and Arauquita.

### Coordinates

6.97 and 7.00 Latitude; -71.76 and -71.61 Longitude.

## Taxonomic coverage

### Taxa included

**Table:** 

Rank	Scientific Name	Common Name
species	*Theobromacacao* L.	Cacao
species	*Carinianapyriformis* Miers.	Abarco
species	*Acaciamangium* Willd.	Acacia
species	*Perseaamericana* L.	Aguacate
species	*Eugeniastipitata* McVaugh.	Arazá
species	*Borojoapatinoi* Cuatrec.	Borojó
species	*Erythrinafusca* Lour.	Bucaré ceibo
species	*Erythrinapoeppigiana* Walp & OF Cook.	Bucaré
species	*Swieteniamacrophylla* King.	Caoba
species	*Cedrellaodorata* L.	Cedro
species	*Pachiraquinata* Jacq.	Ceiba tolúa
species	*Handroanthuschrysanthus* Jacq.	Flor amarillo
species	*Annonamuricata* L.	Guanábana
genus	*Ficus*	Higuerón
species	*Spondiasmombin* L.	Jobo
species	*Leucaenaleucocephala* Lam. & De Wit	Leucaena
species	*Mangiferaindica* L.	Mango
species	*Albiziaguachapele* Kunth & Dugand	Masaguaro - Amusco
species	*Gliricidiasepium* Jacq. - Kunth ex Walp.	Matarratón
species	*Gmelinaarborea* Roxb.	Melina
species	*Citrussinensis*	Naranja
species	*Cordiagerascanthus* L.	Nogal cafetero
species	*Artocarpusaltilis* Parkinson & Fosberg.	Pandeaño
species	*Cordiaalliodora* Ruiz & Pavon	Pardillo
genus	*Musa*	Plátano
species	*Tabebuiarosea* Bertol- Bertero ex A. DC.	Roble
species	*Samaneasaman* Jacq & Merr.	Samán
species	*Tectonagrandis* L.	Teca
species	*Guareaguidonia* C. DC.	Trompillo
species	*Pouroumacecropiifolia* Mart.	Uva caimarona
species	*Matisiacordata* Bonpl.	Zapote

## Temporal coverage

### Notes

Climate and soil temperature data covered the historical period between 1979 and 2020 (Lembrechts 2022). The cacao farms mapped here represented a temporal coverage of approximately 100 years of the Arauca cacao growing region.

## Usage licence

### Usage licence

Creative Commons Public Domain Waiver (CC-Zero)

### IP rights notes

Creative Commons Public Domain Waiver (CC-Zero)

## Data resources

### Data package title

Cacao (*Theobromacacao* L.) climate zones and its associated agrobiodiversity in Arauca, Colombia

### Resource link

https://doi.org/10.5281/zenodo.8302237

### Number of data sets

3

### Data set 1.

#### Data set name

Regional scale

#### Data format

.shp

#### Description

This dataset contains 1,538 occurrences of cacao farms with ID, latitude, longitude, Principal Component Analysis 1 values, municipality name and sampling sites.

**Data set 1.** 

Column label	Column description
ID	Farm Identifier #
Latitude	Geographic Coordinates.
Longitude	Geographic Coordinates.
PCA1	Principal Component Analysis Axis 1 values.
Municipality name	Municipios.
Sampling Sites	Collection sites.

### Data set 2.

#### Data set name

Local scale

#### Data format

.shp

#### Description

This dataset conatins 180 occurrences of cacao farms with ID, municipality, latitude, longitude, climate cluster number.

**Data set 2.** 

Column label	Column description
ID	Farm Identifier #
Municipality	Municipio.
Latitude	Geographic Coordinates.
Longitude	Geographic Coordinates.
Climate cluster #	Number of climate cluster.

### Data set 3.

#### Data set name

Farm scale

#### Data format

.csv

#### Description

This dataset contains georeferenced occurrences of 30 tree species across 180 farms and climate clusters. A .csv file name species_names is attached to this farm-scale dataset.

**Data set 3.** 

Column label	Column description
Code	Farm ID.
ID_geo	Farm ID.
Latitude	Geographic Coordinates.
Longitude	Geographic Coordinates.
Cluster	Cluster number.
Species	Common and scientific name.

## Additional information

Project title in Spanish: Cacao Regalías Arauca or Sistema General de Regalías SGR Cacao Arauca: implementación de estrategias Agroforestales y vinculación de avances en el manejo agronómico y postcosecha de nuevos clones, para mejorar la productividad y calidad del cacao en el departamento de Arauca. This Project is composed of three technical components: agroforestry, cadmium and post harvesting. The farms' occurrence datasets are available for all components.
